# The role of Universal Grammar and crosslinguistic influence in the interpretation of recursive set-subset adjectives in adult Romanian L1-English L2 bilinguals

**DOI:** 10.3389/fnhum.2025.1537488

**Published:** 2025-08-18

**Authors:** Adina Camelia Bleotu, Deborah Foucault, Tom Roeper, Usha Lakshmanan

**Affiliations:** ^1^English Department, Faculty of Foreign Languages and Literatures, University of Bucharest, Bucharest, Romania; ^2^Department of Linguistics, Faculty of Philological and Cultural Studies, University of Vienna, Vienna, Austria; ^3^Department of Linguistics, University of Massachusetts Amherst, Amherst, MA, United States; ^4^Brain and Cognitive Sciences Program, School of Psychological and Behavioral Sciences, Southern Illinois University Carbondale, Carbondale, IL, United States

**Keywords:** recursion, adjective set subset, UG, Romanian L1, English L2, crosslinguistic influence, language transfer

## Abstract

Our research contributes to debates about the role of Universal Grammar constraints and crosslinguistic influence in sequential bilingual acquisition and use. We investigate experimentally how adult Romanian L1-English L2 bilinguals interpret sequential adjectival modifiers of a noun in recursive set-subset contexts in both languages (e.g., *flori mici roşii*, lit. ‘flowers small red’ in Romanian L1, *red small flowers* in English L2, meaning ‘the subset of red flowers among the set of small flowers’, and not the coordinative ‘the red and small flowers’). We ask whether the Recursive Set-Subset Ordering (RSSO) Constraint is observed in both Romanian L1 and proficient English L2 speakers, such that the adjective closer to the head noun indicates the set and the adjective further away indicates the subset. Our study employs a story-based, forced choice comprehension task to test RSSO against Adjective Ordering Restrictions (AORs), as two competing possible sources for adjective ordering and interpretation. While AOR captures ordering preferences of adjectives naming conceptual properties (e.g., A_Size_ A_Color_ N in English, N A_Color_ A_Size_ in Romanian), RSSO posits a structure-dependent principle in terms of sets and subsets (e.g., A_Subset_ A_Set_ N in English, N A_Set_ A_Subset_ in Romanian). We find that bilinguals adhere to the RSSO in both languages even in contexts where AOR and RSSO are in conflict. This finding supports RSSO’s status as a UG syntactic-semantic constraint. Interestingly, for a few participants, we also found evidence for crosslinguistic influence stemming from language-specific differences in branching directionality, linear order, and AORs.

## 1 Introduction

Since the early stages of research within the field of bilingualism, an important goal has been to shed light on the representation and processing of two languages within the bilingual mind. Debates continue to center around whether the two languages of a bilingual have a shared or a separate representation (Kroll and Tokowicz, 2009). Furthermore, there is ongoing discussion about whether each language is processed independently of the other when bilinguals are in a primarily “monolingual mode” as when they are actively communicating using only one of their two languages, with the other language essentially deactivated (Grosjean, 2010). Bilingual language processing is modulated by various factors such as the following: typological similarities and differences between the two languages, age at onset of exposure, contexts of acquisition (simultaneous versus sequential; immersion versus formal instructed learning), frequency of use, level of proficiency, and language dominance (Grosjean, 2010). A number of studies using different methodologies suggest that there are both shared and separate representations. Findings from the literature support a shared system at the semantic or conceptual level corresponding to word meaning (Francis, 2009) but separate representations at the lexical level corresponding to word form (Kroll and Stewart, 1994; Kroll et al., 2010; Costa, 2009). As for syntax, some studies have found evidence for a shared syntactic representation, particularly in relation to constructions which are functionally and/or structurally similar across the two languages (e.g., Hartsuiker et al., 2004; Loebell and Bock, 2003; Salamoura and Williams, 2006, 2007; Shin and Christianson, 2009). Other studies have provided evidence favoring separate representations, particularly for constructions that are structurally dissimilar as well as in contexts where the language mode is primarily a monolingual one (e.g., Ahn and Ferreira, 2024; Bernolet et al., 2007; Kim and McDonough, 2008).

A related issue concerns the role of *crosslinguistic influence*. Traditionally, influence of one language upon the other has been framed in terms of *transfer* (i.e., language transfer). Note that Uriel Weinreich (1953) in his seminal book on bilingualism viewed language transfer as potentially bidirectional, with L1 and L2 influencing one another. However, until very recently, most second language acquisition research focused on L1 transfer, i.e., unidirectional transfer where the L1 influences the developing L2 (Gass and Selinker, 2008). Recent research on simultaneous and/or sequential bilingual language development has shown that interaction among the languages is not solely unidirectional from L1 to L2 but bidirectional (Cook, 2003; Pavlenko, 2004; Pavlenko and Jarvis, 2002; Schmid and Köpke, 2017). In the present study, we prefer the term “crosslinguistic influence,” as it is a broader term that encompasses any form of interaction between the languages in a bilingual’s (or multilingual’s) repertoire. Crosslinguistic influence can manifest as either facilitation or interference. On the one hand, when facilitative it can support and enhance the development of the target language. On the other hand, it can result in interference, as when activation of properties from one language hinders the processing and acquisition of another. Furthermore, the term “language transfer,” which also stems from the behaviorist research paradigm, has recently been criticized (see Sharwood Smith, 2021) as a pernicious notion, misrepresenting how linguistic properties function in the mind. He suggests that, instead of transfer, languages are interconnected within a shared cognitive system, where structures coactivate without transferring properties. Furthermore, we concur with Berkes and Flynn (2016) that language learners do not build the new grammar by merely transferring the language-specific features of the L1 into the new language. Instead, they construct the new grammar via *grammatical mapping* as in the case of L1 acquisition (Lust, 2012). In other words, what learners do is “map from one primary structure to a more developed structure by dissociating modular grammatical components and integrating them in the ‘assembly’ of new language-specific grammars” (Flynn et al., 2005:2, cited in Berkes and Flynn, 2016; see also Flynn et al., 2004; Fernández-Berkes and Flynn, 2023; Lakshmanan, 2024; Lust et al., 2025). The primary structure is a minimalist Universal Grammar (UG), a fundamental and an invariant template guiding the mapping to a language-specific grammar. When acquiring a second or third language, the previously acquired language/s can cumulatively enhance the process of grammatical mapping to the language-specific grammar. Merge, a core UG operation at the heart of grammar building, provides the template for combining syntactic elements (e.g., words, phrases, clauses, sentences) into recursive hierarchical structures (e.g., coordination, adjunction, and embedding, etc.), enabling the unbounded expressive power of language.

With growing interest in language maintenance and attrition (in particular, L1 attrition), increasing attention has been given to how subsequent languages influence the L1. Existing research indicates that all linguistic subsystems, phonology, morphology, lexis, syntax, semantics and conceptual representation, as well as pragmatics, are susceptible to varying degrees of crosslinguistic influence, with certain aspects (e.g., lexis) being more vulnerable than other aspects, such as syntax (Gallo et al., 2021; Gürel, 2008; Schmid and Köpke, 2017; Lakshmanan, 2024; Teruya and Lakshmanan, 2019).

Historically, second language acquisition research has compared L2 learners to monolingual speakers of the target language in order to determine L2 learners’ success in converging upon L1 (native)-speaker norms for the target L2. This approach tends to emphasize the failure of L2 learners to achieve the same level of competence as L1 speakers.^1^ As Grosjean (2010) has cautioned, a bilingual is *not* two monolingual speakers in the same mind/person. Furthermore, Cook’s (1991, 2002, 2012) multicompetence framework, which assumes that language transfer, i.e., crosslinguistic influence, is potentially bidirectional, holds that it would be erroneous to expect bilingual speakers to function exactly like monolingual speakers in each of their two languages. Furthermore, Falk and Bardel (2010) emphasize that an L2 can influence an L3 to a degree comparable to that of an L1, both at the lexical and syntactical levels. However, they also note that it is often unclear which specific background language—whether L1 or L2—plays a more decisive role in the crosslinguistic influence on an L3. This is further supported by scholars who have argued in favor of the cumulative enhancement model, according to which all languages known can potentially influence the development of subsequent learning (e.g., Flynn et al., 2004). A substantial body of research on bilingual code-switching has shown that bilinguals are able to code-switch between their two languages with ease when they are actively using both languages simultaneously, i.e., when they are in a bilingual mode. At the same time, even when only one language is in use, as when they are in a monolingual mode, the other language that is not in use is also “activated.” Such crosslanguage or parallel activation during a monolingual mode has been observed for different aspects of language from lexis to syntax. When the activation involves elements or aspects that are not shared across the two linguistic systems, inhibition would need to occur in order to ensure convergence upon the target language in use (Bialystok, 2001; Green, 1998; Grosjean, 2010; De Groot and Kroll, 1997).

Generative approaches to bilingualism and second language acquisition seek to determine in what way the innate biological endowment for language, that is the invariant and parameterized principles of Universal Grammar (UG), remains available across the two languages of a bilingual. It is expected that invariant principles of UG such as *structure dependency* will be accessible for acquisition and use of a subsequent language. This means that an invariant principle of UG would be instantiated in the same way across L1 and L2. However, even though we expect that both languages will adhere to an invariant UG constraint, it is possible that its operation could be impacted by other cognitive or linguistic properties or processes. *Structure dependency* is a fundamental property of human language (see for instance, Chomsky, 1971, 1980), according to which grammatical operations are based upon the hierarchical phrasal structure rather than upon a linear sequence of first, second, third, etc. It has been shown that acquisition relies on abstract structural principles rather than on a linear ordering of words (Crain and Nakayama, 1987; Berwick et al., 2011). However, in real time processing, parsing strategies (like those proposed by Fodor and Frazier, 1978 and De Vincenzi, 1991) can interact with structure dependency. Assuming that a UG principle is parameterized, as for example in relation to *Head directionality* (i.e., head-first versus head-last) or *branching directionality* (i.e., right-branching versus left-branching phrase structure),^2^ the expectation is that acquisition and use of a new language will be easier in instances where the parametric setting or value is the same for both languages.^3^ In those instances where the parameter setting is different for each language, particularly in relation to sequential bilinguals, the L1 (or the dominant language), could impact the use of the L2. “Resetting” of the UG parameter (or more appropriately, activation of the L2 parametric value) would need to occur based on positive evidence, as well as in cases where the input is not sufficiently rich or precise (poverty of stimulus). The existing evidence in second language acquisition has been largely consistent with the *strong continuity hypothesis*, which holds that UG continues to remain fully available for subsequent language acquisition (Epstein et al., 1996, 1998; Flynn, 1987; Lakshmanan, 1994; Lardiere, 1998; White, 2003; Schwartz and Sprouse, 1996; among others). Continued access to UG, however, does not entail the rejection of L1 influence. Various proposals have been put forth in L2 research on the role of L1 transfer: *No* L1 transfer, *Partial* L1 transfer and *Full* L1 transfer (for review, see White, 2003; see also Pienemann et al., 2005 for discussion of psycholinguistic processing constraints on L1 transfer). As discussed previously, along with Berkes and Flynn (2016), we assume that second language learners do not construct the new grammar by mere transference of language-specific features from the L1 into the new language. Rather, as in the case of L1 acquisition, they build the new grammar via a process of *grammatical mapping*, which involves mapping from one primary structure (i.e., a minimalist UG-determined template) to a more developed structure. This involves a process of dissociation and integration of modular grammar components in constructing the new language-specific grammar/s, despite the wide variation both within and across languages (e.g., branching directionality/Head-directionality parameter, tense and agreement morphology, null arguments, associated semantics and pragmatics, etc.). As mentioned previously, in the case of second and third language acquisition, all previously acquired language specific grammars have the potential to cumulatively enhance the mapping from the initial UG template to the new language-specific grammar. In relation to real time processing of phrases and sentences, due to potential cross-language activation, in cases where the L1 and L2 differ, the processing of a new language for comprehension and production during a monolingual language mode could involve the inhibition of an L1 property or parametric value and activation of the appropriate L2 property or value.

In the current paper, we investigate the acquisition and use of recursive adjectives in an adult sequential bilingual context. Our focus here is on L1 Romanian-L2 English bilinguals who began learning English in a formal instructional setting in Romania as a foreign language during early childhood and were pursuing their undergraduate studies at a university in Romania. Importantly, our goal is to investigate how highly proficient English L2 users with Romanian as the L1 and their dominant language interpret complex recursive adjective phrases in both their languages. Our main focus is on their knowledge and use of these structures in both their L1 and L2. In this study, we test the universality of the Recursive Set Subset Ordering (RSSO) Constraint, a UG-based structure-dependent principle according to which set adjectives are merged to the head noun first, and subset adjectives are merged subsequently. Specifically, our study provides novel evidence from English, a language with left-branching adjective structures, and contrasts it with Romanian, a language with right-branching adjective structures. By comparing recursive modification across these languages, we aim to assess whether branching directionality, as well as other cognitive factors—such as Adjective Ordering Restrictions (AOR) in terms of conceptual properties (like size or color)—modulate adherence to the RSSO constraint. In so doing, the study contributes to our understanding of how universal grammatical constraints interface with language-specific variation and other cognitive factors in the L1 and L2 comprehension of bilinguals.

## 2 Background on adjectives and adjective orders in English and Romanian

From a global perspective, and in terms of Principal Branching Directionality (Lust, 1983; Lust and Mangione, 1983), both English and Romanian are head initial, right branching languages. However, they differ in their branching directionality at the local phasal level (Lakshmanan et al., 2024). For example, adjectival phrases in English are left branching (prenominal) while in Romanian they are right branching (postnominal), as shown in (1) and (2) respectively. English is a natural gender language and Romanian is a language with grammatical gender, where adjectival modifiers *agree* both in number and gender with the head noun.

(1) a. big red flowers
       b. red big flowers      (English)
(2) a. flori    roşii    mari
         flowers red-F.PL big-F.PL
         ‘big red flowers’
       b. flori    mari    roşii
         flowers big-F.PL red-F.PL
         ‘red big flowers’      (Romanian)

Both languages allow two different interpretations: (i) a coordinative interpretation, and (ii) a recursive set-subset interpretation. For instance, under a coordinative interpretation for (1a), the phrase *big red flowers* refers to flowers that are both big and red. Here, the two adjectives, which are not in a set-subset relationship, are combined together first before the resulting unit is merged with the head noun. In contrast, under a recursive set-subset interpretation, the same phrase refers to a subset of big flowers from a set of red flowers. The same is true of the corresponding Romanian adjectival phrase in (2a). As for the phrase *red big flowers* in (1b), under a coordinative interpretation, it refers to flowers that are both red and big. In contrast, under a recursive set-subset interpretation, the same phrase refers to a subset of red flowers from a set of big flowers. This is true of the corresponding Romanian adjectival phrase in (2b).^4^ Note that regardless of whether the adjectives occur prenominally (as in English) or postnominally (as in Romanian), the adjective that specifies the set must be closest to the head noun, and the adjective that specifies the subset is further away from the head noun. This universal constraint at the syntax-semantic interface is known as the *Recursive Set Subset Ordering Constraint* (Foucault, 2020; Foucault et al., 2021, 2022a; Bleotu and Roeper, 2021a, 2021b, 2022a, 2022b), as defined in (3), and it constitutes the focus of the current study:

(3) *The Recursive Set-Subset Ordering Constraint* (RSSO): In a context requiring identifying a subset within a set, the adjective specifying the set is merged to the noun first, with the subset adjective merged subsequently.

In addition to the *Recursive Set Subset Ordering Constraint* (RSSO) on adjectives, there is also an Adjective Ordering Restriction (AOR) on the linear sequence of adjectives in language, associated with coordinative (non-set-subset) uses of adjectives, as in *big red flowers* understood as ‘big and red flowers’. Across many languages in the world, it has been argued that more objective properties, such as color, appear closer to the noun, whereas more subjective properties, such as size, appear further away from the noun (Dixon, 1982; Sproat and Shih, 1991; Scontras et al., 2017, 2019). This is illustrated in (4):

(4) QUALITY > SIZE> SHAPE > COLOR > PROVENANCE (Sproat and Shih, 1991)

AORs have given rise to debates regarding their status (universal vs. language-specific property) and their nature (syntactic/semantic/cognitive). According to Cinque’s (1994, 2005, 2023) cartographic approach, AORs are syntactic universals subject to crosslinguistic variation. Apart from the fact that adjectives vary in how they are ordered with respect to the noun (prenominal vs. postnominal), adjectives also vary in how they are ordered with respect to one another. For instance, in a Germanic language like English, where adjectives are prenominal, the most natural order for a sequence of adjectives involving a Size adjective and a Color adjective is A_Size_ A_Color_ N. In contrast, in a Romance language like Romanian, where adjectives are postnominal, the most natural order for such a sequence would be N A_Color_ A_Size_, which represents a mirror order of English. It is important to note that the AORs apply to coordinative contexts and not to recursive set-subset contexts.

For the purposes of the current paper, we assume that AORs involve a strict hierarchy across languages, in line with Cinque and others. However, it is relevant to mention that, for Romanian, some recent studies have suggested a less rigid AOR ordering (see e.g., Cornilescu and Cosma, 2019; Bleotu and Luciu, 2024; Trușcă and Bleotu, 2024, see also Leivada and Westergaard, 2019 for Greek). In coordinative contexts, native speakers of British English tend to observe AORs overall, e.g., the order A_Size_ A_Color_ N (e.g., big red flowers) is overwhelmingly preferred over A_Color._ A_Size_ N (e.g., red big flowers). In contrast, in Romanian, besides the expected mirror order of English N A_Color_ A_Size_ (e.g., flowers red big), the order N A_Size_ A_Color_ (e.g., flowers big red) has also been reported. It is possible that the emerging flexibility recently observed in Romanian could stem from the influence of English, given that today in Romania, L1 Romanian speakers learn and use English regularly. Therefore, more studies are needed to conclusively shed light on the issue. In our study, we assume along with Cinque, that AORs are in principle observed in Romanian as well, with the understanding that there may be some flexibility, especially in the case of bilinguals (for discussion on the influence of English on Romanian, see, for instance, Avram, 1997; Stoichiţoiu-Ichim, 2001, 2002, 2003).

**Figure 1:**
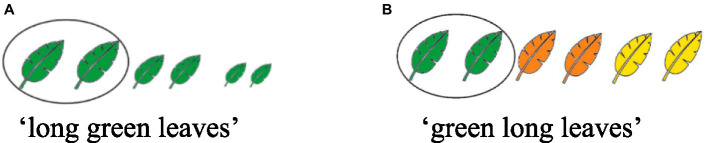
Pictures employed in Bleotu and Roeper (2022a, 2022b).

Moreover, and most importantly, recent research with adults and children shows that, in recursive set-subset contexts, the universal RSSO constraint overrides AORs in English, as well as in Romanian. One line of research, exemplified by Foucault et al. (2021, 2022a) and Bleotu and Roeper (2021a, 2021b), which build upon on Roeper and Snyder (2004) and Roeper (2011), looked at recursive adjectives such as *small big flowers* and *big small big mushrooms*, adjectives which involve gradable properties characterizing the same dimension (size). Such structures typically give rise to recursive set-subset interpretations because coordinative readings (e.g., *small and big*) involve contradictory properties and are therefore less plausible. In such recursive interpretations, the outer adjective modifies the property introduced by the inner adjective, reflecting a set-subset hierarchy. For instance, in a phrase like *a large small shirt*, *large* is interpreted relative to the category *small shirt* rather than as a simple conjunction of *large* and *small*. Another line of research involves adjectives which specify properties characterizing different dimensions (size, color): *long green leaves* or *green long leaves.* Such adjectives can give rise not only to recursive set-subset interpretations but also to conjunctive readings, without a set-subset hierarchical relationship. However, in contexts where a clear set-subset distinction is present—such as visually salient subsets—speakers tend to order adjectives to reflect this hierarchy. Bleotu and Roeper (2022a, 2022b) tested the validity of the *Recursive Set-Subset Ordering Constraint* (RSSO) as defined in (3) above, in order to see whether participants observe RSSO or AOR for such sequences in recursive set-subset contexts. In a forced choice task, participants had to choose the best answer to describe a picture. The authors found that, when describing a picture depicting a subset of long leaves within a set of green leaves (see Figure 1A), Romanian adults preferred to merge the Color adjective before the Size adjective (5a). Conversely, when referring to green leaves within a set of long leaves (see Figure 1B), they preferred merging the Size adjective first to the head noun (5b). The order *frunze verzi lungi* ‘leaves green long’ is not in line with the strict SizeA ColorA Noun AORs predicted for Romanian by Cinque’s approach. According to Cinque (1994, 2005), in coordinative contexts, the order for English should be SizeA ColorA N, while for Romanian, the mirror order Noun ColorA SizeA should hold. Interestingly, children and adults tend to choose N ColorA SizeA orders if the color adjective picks the set and the size adjective picks the subset, but they tend to choose N SizeA ColorA orders if the size adjective picks the set and the color adjective picks the subset. Their empirical findings confirmed that participants will order adjectives according to a set-subset hierarchy over AORs when the context requires it.

(5) a. *frunze verzi       lungi*
        leaves green-F. PL long-F. PL
        ‘long green leaves’
      b. *frunze lung       verzi*
        leaves long-F. PL green-F. PL
        ‘green long leaves’

This study and others bring supporting evidence that monolingual Romanian and American English children and adults observe the by RSSO by choosing to associate the adjective merged closest to the head noun with a set interpretation and the adjective merged further away from the head noun with a subset interpretation, regardless of the adjective employed (size/color) (for Romanian data, see Bleotu and Roeper, 2021a, 2021b; for American English data, see Foucault et al., 2021, 2022a,b, 2024). Moreover, this seems to be the case for bilingual English-speaking children as well (see Lakshmanan et al., 2022, 2024). Crucially for our current study on adult Romanian-English sequential bilinguals, it is relevant to mention that Foucault et al. (2022a) found that, in set-subset contexts, American English L1 adult controls observe RSSO with very high accuracy, producing recursive set-subset adjective phrases involving gradable size adjectives (*small big mushrooms*) and interpreting these recursively.

The current study extends the aforementioned research on the syntactic-semantic interface of RSSO as a fundamental UG principle to a bilingual context. Given that the RSSO is a syntactic constraint, there are two possible analyses of recursive set-subset adjectives: an adjunction analysis and a cartographic analysis. According to the adjunction analysis (Kremers, 2003; Abels and Neeleman, 2010), languages differ based on branching directionality (Lakshmanan et al., 2024; Lust, 1983; Lust and Mangione, 1983). From a local perspective within this view, English places adjectives to the left of the noun, whereas Romanian positions them to the right. One could postulate that the RSSO is a further requirement within Adjunction, merging set adjectives to head noun first. In a cartographic approach (Cinque, 1994, 2005, 2010), the underlying UG default would correspond to the English order Subset Set Noun, and the Romanian Noun Set Subset surface order, which is a mirror order of English, would be derived via movement. In particular, it could be achieved through a complex set of operations known as Roll-Up-of-N, which manipulates the basic English adjectival sequence through successive NP movement through specifiers of functional projections. The analyses are illustrated for Romanian in (6).

**Figure:**
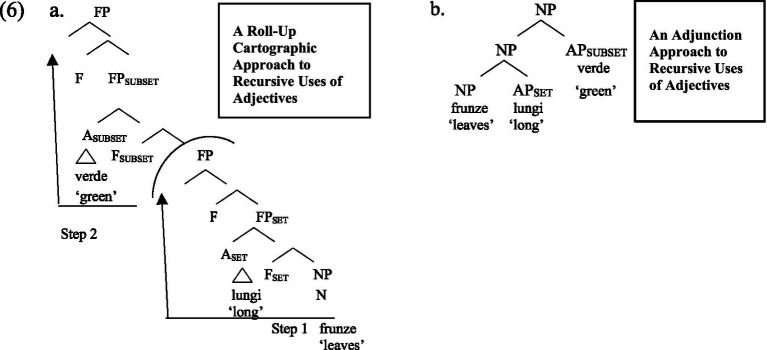


Note that, in example (6a), the noun phrase (NP) moves from its original position to an outer specifier of FP_set_ (Step 1). Then, the newly formed FP, containing FP_set_, moves again to the outer specifier of the projection hosting FP_subset_ (Step 2). Deciding between an Adjunction account of RSSO and a Roll UP Account of RSSO is not trivial, and, for this reason, we abstain from committing to either. However, what is important for the current study is that both English and Romanian observe RSSO in recursive set-subset contexts. This is supported by ample empirical evidence from both adults and children speaking these languages as L1.

## 3 Background on the acquisition and use of recursive structures in bilinguals

Compared to studies on monolinguals, there are relatively few studies on bilingual children’s development of recursive structures. The few studies that have compared bilinguals and monolinguals suggest that they handle recursion similarly. For instance, Pérez-Leroux et al. (2017) and Pérez-Leroux et al. (2021) compared the recursive prepositional phrases (PPs) produced by 4- to 6-year-old Spanish-English bilinguals and monolingual English speakers. They found that bilinguals performed similarly to monolinguals with 2- and 3-level PP-recursion (e.g., *the dog next to the tree next to the house*). Avram et al. (2020) found that 5- to 7-year-old simultaneous Romanian-Hungarian bilinguals had the same acquisition path as their monolingual Romanian children for recursive PPs and relative clauses. Recent studies by Foucault et al. (2024) and Lakshmanan et al. (2022, 2024) found that both monolingual and bilingual American English-speaking children can comprehend and produce recursive possessives and recursive gradable adjectives (*big small mushrooms, small big small mushrooms*) from as early as age 4, with children between 4 to 6 experiencing more challenges than children between 7 to 12. Both groups found recursive adjectives more challenging than recursive possessives, but their overall mastery was quite high with both. Interestingly, Leandro and Amaral (2014) observed that bilingual Wapichana-English children outperformed monolinguals (up to the age of 7) in interpreting complex genitive constructions, supporting the idea of a bilingual advantage. These findings support the notion that recursion is an innate feature of Universal Grammar.

However, the few studies that have examined the acquisition of recursion by adult L2 speakers showed that they experienced some challenges in comparison to L1 monolingual speakers. For example, Limbach and Adone (2010) found that, although L1 German-L2 English speakers who had acquired English in adulthood were equally successful as L1 English-speaking children in their comprehension of recursive possessives, they were not as successful as L1 English-speaking adults. Nelson (2016) investigated the comprehension of recursive PPs in sequential bilingual adult speakers (L1 English-L2 Spanish, L1 Spanish-L2 English). The study found that while adult L2 speakers experienced more challenges in comprehension than monolinguals, their accuracy with recursion improved with increasing L2 proficiency.

Besides comparing monolingual and bilingual children’s ability to handle recursive phrases, Lakshmanan et al. (2024) investigated whether branching directionality (Left, Right, and Mixed) of nominals in the bilingual children’s non-English language influences their comprehension and production of left-branching recursive possessives and gradable adjectives in English, their stronger language. They assessed 1-level, 2-level, 3-level recursive adjectives, and found that the bilingual children in the Left-branching group (e.g., Mandarin Chinese, Hindi, Kannada) had the lowest accuracy scores, even though their other language matched the branching directionality of the target recursive phrases in English. The Mixed-branching group (e.g., German, Russian) had the highest accuracy scores on all except 3-level recursive adjectives (e.g., *small big small mushrooms*), for which the Right-branching group (e.g., Spanish, French) had slightly higher scores. However, the differences in accuracy scores between the three groups were not statistically significant, suggesting that further research is needed involving a larger sample size, as well as assessment of both languages of the bilinguals, and not just the stronger one.

Other relevant research on bilinguals focuses on how adjectives are linearly ordered with respect to the noun they modify. Some studies on L2 Spanish indicate that, regardless of L1-L2 differences in the linear ordering of the adjective and noun, advanced learners are able to successfully comprehend and produce the target L2 Spanish orders (Judy et al., 2008; Guijarro-Fuentes et al., 2009; Rothman et al., 2009, 2010). However, Nicoladis (2006) found that L1 French-L2 English preschool bilingual children experienced challenges with the English A N order, which differs from the predominant N A order in their French L1. Interestingly, in cases where the two languages are both Romance languages (e.g., French and Spanish) with predominantly postnominal adjectives, Androutsopoulou et al. (2008) found that French L1-Spanish L2 adult speakers experienced challenges with evaluative adjectives occurring prenominally in Spanish but postnominally in French (e.g., *una fantástica película* in Spanish / *un fantastique film in French “a fantastic film”).

## 4 The current study: aims and predictions

As stated in the introduction, our study examines how adult Romanian L1-English L2 bilinguals with high level of proficiency in English L2 comprehend recursive set-subset adjectives in L1 (Romanian) and L2 (English). These two languages differ in branching directionality of adjectival modifiers of a head noun: English is prenominal (left-branching), while Romanian is postnominal (right-branching). Consequently, in set-subset contexts, Romanian is a mirror image of English: *red small flowers* is *flori mici roşii* ‘flowers small red’. Our study thus constitutes a direct test of RSSO against AOR, as two competing possible sources for adjective ordering and interpretation. While AOR captures ordering preferences of adjectives naming conceptual properties (e.g., size-before-color in English, color-before-size in Romanian), RSSO posits a universally determined, structure-dependent principle, merging set adjectives with the head noun first, and subset adjectives subsequently. By examining recursive adjective structures in both Romanian (right-branching) and English (left-branching), we take advantage of the fact that the two languages instantiate opposite surface linear orders, and we investigate whether they observe the same underlying structural constraint (RSSO). This crosslinguistic comparison allows us to test whether RSSO overrides AOR, even when the surface order is reversed due to branching differences. By observing whether bilinguals adhere to RSSO across languages despite different surface orders, we probe the universality and dominance of structure-dependent mechanisms in linearization, and their interaction with crosslinguistic influence and processing constraints.

According to the Recursive Set Subset Ordering Constraint, set adjectives are merged first to the head noun, and subset adjectives are merged subsequently. Given that RSSO is a UG principle, our prediction regarding its availability is as follows:

(7) *UG Prediction*: Romanian-English bilinguals will adhere to RSSO in both their L1 and L2.

This means that when the context is a recursive set/subset context, participants will observe the RSSO constraint by providing the target recursive interpretation and not a coordinative interpretation, regardless of whether the linear order of adjectives is congruent or incongruent with AOR. For example, in a recursive context where the size adjective *small* represents the set and the color adjective *red* represents the subset, participants will interpret *red small flowers* in English and *flori mici roşii ‘flowers small red’* in Romanian recursively (as ‘the subset of red flowers within the set of small flowers’) and not coordinatively (as ‘the red and small flowers’). It is important to mention that, because the coordinative interpretation may generally be more frequent, it is possible that the representation associated with the coordinative interpretation would be activated and would need inhibition in both L1 and L2.

At the same time, we acknowledge potential for crosslinguistic influence. Whether this influence is unidirectional or bidirectional depends on the proficiency level and relative dominance of the bilinguals’ L1 and L2. Recall that in relation to adjectival modifiers of the head noun, Romanian is right-branching and English is left-branching. Thus, we expect that crosslinguistic influence could be triggered in several ways. One possible trigger is the simultaneous activation of the branching directionality of both English L2 and Romanian L1 even when only one language is in use. Another possibility is that the crosslinguistic influence could be triggered by the linear ordering of recursive adjectives (from left to right), regardless of the noun: English has the order A_Subset_ A_Set_ Noun, whereas Romanian has the order Noun A_Set_ A_Subset_. Given that in the linear ordering, the subset adjectival modifier precedes the set in English, but in Romanian the set precedes the subset, the reverse order may be activated and, if not inhibited, would lead to the non-target interpretation.

In our study, we focus on adult L1 Romanian-L2 English sequential bilinguals who are highly proficient in their L2. We formulate the following prediction in relation to crosslinguistic influence:

(8) *Prediction for Crosslinguistic influence:* Given that the dominant language of participants is Romanian, and that they use it more frequently than English, we expect a greater likelihood for Romanian to influence their English than for their English to influence Romanian.

Specifically, even when only English is in use, the simultaneous crosslanguage activation of the left-branching directionality of English, as well as of the right-branching directionality of Romanian, could lead to errors with recursive structures in English because of failure to inhibit the more dominant L1. At the same time, given the high L2 proficiency of bilinguals, we cannot reject the potential for English to influence Romanian.

Importantly, another possibility also arises. In light of the competence-performance distinction, it may also be that recursion is well in place but handling it in real time may pose challenges because of performance related issues: for example, inhibiting L1 influence especially when there are time constraints.

## 5 Materials and methods

The study received approval from the Research Ethics Committee at the University of Bucharest (59/17.02.2022), and participants provided informed consent prior to data collection. The participants were 41 Romanian L1 sequential bilinguals with C1 English^5^: 21 were tested in their L1 (L1 Romanian group), 20 in their L2 (English L2 group, age range: 18–19, 18 Female; 2 Male mean age: 18; 06).^6^ According to the European classification system, C1 learners are advanced users who can understand a wide range of long, complex texts. They can interact and express themselves fluently and spontaneously. All participants were undergraduate students at the University of Bucharest. They were exposed to English L2 from around age 4–5 and were subsequently exposed to it formally in an instructional setting at least two times a week for 2–6 h (from primary school to university). All the participants we tested studied Romanian as a major and English as a minor at the university. Importantly, we tested the two groups of participants independently: one group was assessed in their L1, and the other group was assessed in their L2. This was done to facilitate a monolingual mode for assessment purposes, as well as to avoid a possible priming effect or bias: if the same participant had been tested on both languages, they might have ended up producing more non-target answers simply given the co-activation of two grammars on the same structures. The groups were matched in terms of level of English, exposure to English and frequency of exposure, age range. Moreover, it is important to reiterate that previous research on recursion in bilinguals tended to assess only one language (typically L2), whereas in the current research, both L1 and L2 are assessed.

After providing informed consent, participants completed a story-based, forced choice comprehension task (Tables 1, 2). The task began with 2 practice trials to familiarize them with the task methodology. The experiment itself contained 8 test items^7^ that were interspersed with 4 fillers.^8^ The test items were designed to examine whether participants exhibited a preference for recursive adjectival orders in line with the RSSO (N SetA SubsetA in Romanian, SubsetA SetA N in English) or whether they have a preference for adjective orders that align with Adjective Ordering Restrictions (AOR) in terms of conceptual properties (like Color or Size), as predicted by Cinque (1994, 2010). Recall that AOR holds for both left- and right-branching structures. Thus, according to Cinque, AOR in Romanian (a postnominal adjective language) should mirror the order found in English (a prenominal adjective language). The 8 test items (see Table 1) involved 4 items containing recursive adjectival orders complying with AORs and 4 items containing recursive adjectival orders that are incongruent with AORs.

**Table 1:** Adjective-modified nouns tested in the current study.

Items	Congruent with AOR	Incongruent with AOR
*frunze* ‘leaves’	*frunze galbene scurte* leaves yellow-F. PLshort-F. PL ‘short yellow leaves’	*frunze lungi verzi* leaves long-F. PL green-F. PL ‘green long leaves’
*leaves*	*short yellow leaves*	*green long leaves*
*flori* ‘flowers’	*flori albastre mari* flowers blue-F. PL big-F. PL ‘big blue flowers’	*flori mici roşii* flowers small-F. PL red-F. PL ‘red small flowers’
*flowers*	*big blue flowers*	*red small flowers*
*veveriţe* ‘squirrels’	*veverite maro grase* squirrels brown-F. PL fat-F. PL ‘fat brown squirrels’	*veveriţe slabe portocalii* squirrels thin-F. PL orange-F. PL ‘orange thin squirrels’
squirrels	*fat brown squirrels*	*orange thin squirrels*
*giraffe* ‘giraffes’	*girafe maronii înalte* giraffes brown-F. PL tall-F. PL ‘tall brown giraffes’	*girafe scunde galbene* giraffes short-F. PL yellow-F. PL ‘yellow short giraffes’
giraffes	*tall brown giraffes*	*yellow short giraffes*

**Table 2:** Examples of the story-based task used to elicit the bilingual participants’ comprehension of recursive adjectives in Romanian-L1 and English-L2.

Congruent with AOR Romanian: N ColorA SizeA English: SizeA ColorA N	Incongruent with AOR Romanian: N SizeA ColorA English: ColorA SizeA N
Romanian	
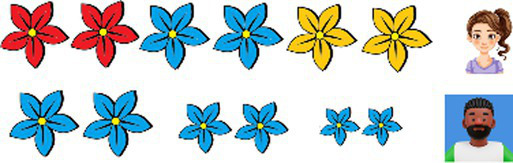 Kim și Paul au grijă fiecare de florile din grădina lor. În grădina lui Kim, Kim are grijă de florile mari. În grădina lui Paul, Paul are grijă de florile albastre. Pe cine ar trebui să rugăm să vedem *florile albastre mari*? Kim (Are flori mari) Paul (Are flori albastre)	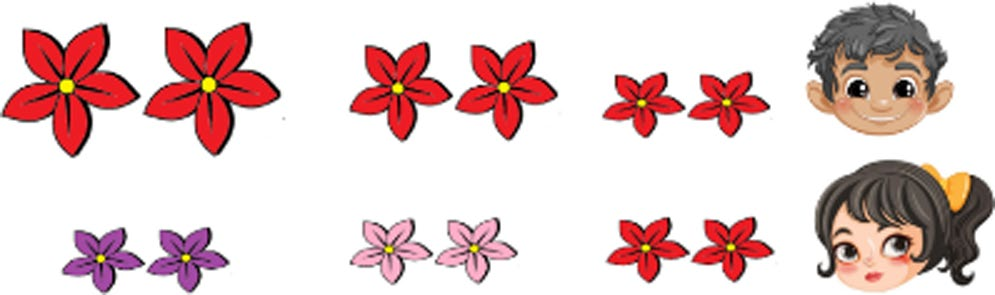 Deb și Aris se ocupă amândoi de flori. În grădina lui Deb, Deb are grijă de florile mici. În grădina lui Aris, Aris are grijă de florile roșii. Pe cine ar trebui să rugăm să vedem *florile mici roșii*? Deb (Are flori mici) Aris (Are flori rosii)
English	
Kim and Paul each take care of flowers. In Kim’s garden, Kim takes care of big flowers. In Paul’s garden, Paul takes care of blue flowers. Who should we ask to see *the big blue flowers?* Kim (She has big flowers) Paul (He has blue flowers) Expected recursive answer: *Paul*	Deb and Aris each take care of flowers. In Deb’s garden, Deb takes care of small flowers. In Aris’s garden, Aris takes care of red flowers. Who should we ask to see *the red small flowers?* Deb (She has small flowers) Aris (He has red flowers) Expected recursive answer: *Deb*

In the case of **AOR-congruent items**, adjectives followed the expected AORs. In English, this means SizeA ColorA N (e.g., *the small red flowers*), while in Romanian, this is the mirror order N ColorA SizeA (e.g., *florile roșii mici*, ‘flowers-the red small’).

In the case of **AOR-incongruent test items**, adjectives appeared in a reverse order, going against AOR. In English, this means ColorA SizeA N (e.g., *the red small flowers*), while in Romanian, this corresponds to N SizeA ColorA (e.g., *florile mici roșii*, ‘flowers-the small red’).

Importantly, prosody-wise, the items were presented to our participants with a neutral intonation throughout, with no contrastive stress or special emphasis on either of the adjectives. This was done to ensure that any interpretative preferences observed were not influenced by prosodic focus or stress.

Participants were introduced to two characters who each had a set of objects. Each set contained 3 subsets of 2 objects: the first character had objects of the same color but varying in size, while the second character had objects of the same size but varying in color. Participants then indicated who had the target subset/s (e.g., *small red flowers*). Table 2 exemplifies the procedure used for both Romanian L1 and English L2. Further consult Supplementary materials for examples of practice items and fillers.

This design specifically pits the structure-dependent RSSO against the AOR (e.g., English’s preference for the linear order size-before-color, Romanian’s preference for the linear order color-before-size). If in the set-subset context of our experiment bilingual participants consistently provide recursive set-subset interpretations in line with RSSO across both L1 and L2, regardless of language-specific AORs, this supports the hypothesis that the set-subset structure-dependent constraint guides interpretation more robustly than AORs. Conversely, deviations from this pattern—especially in L2—can provide evidence for processing difficulties in inhibiting language-specific features.

## 6 Results

**Figure 2:**
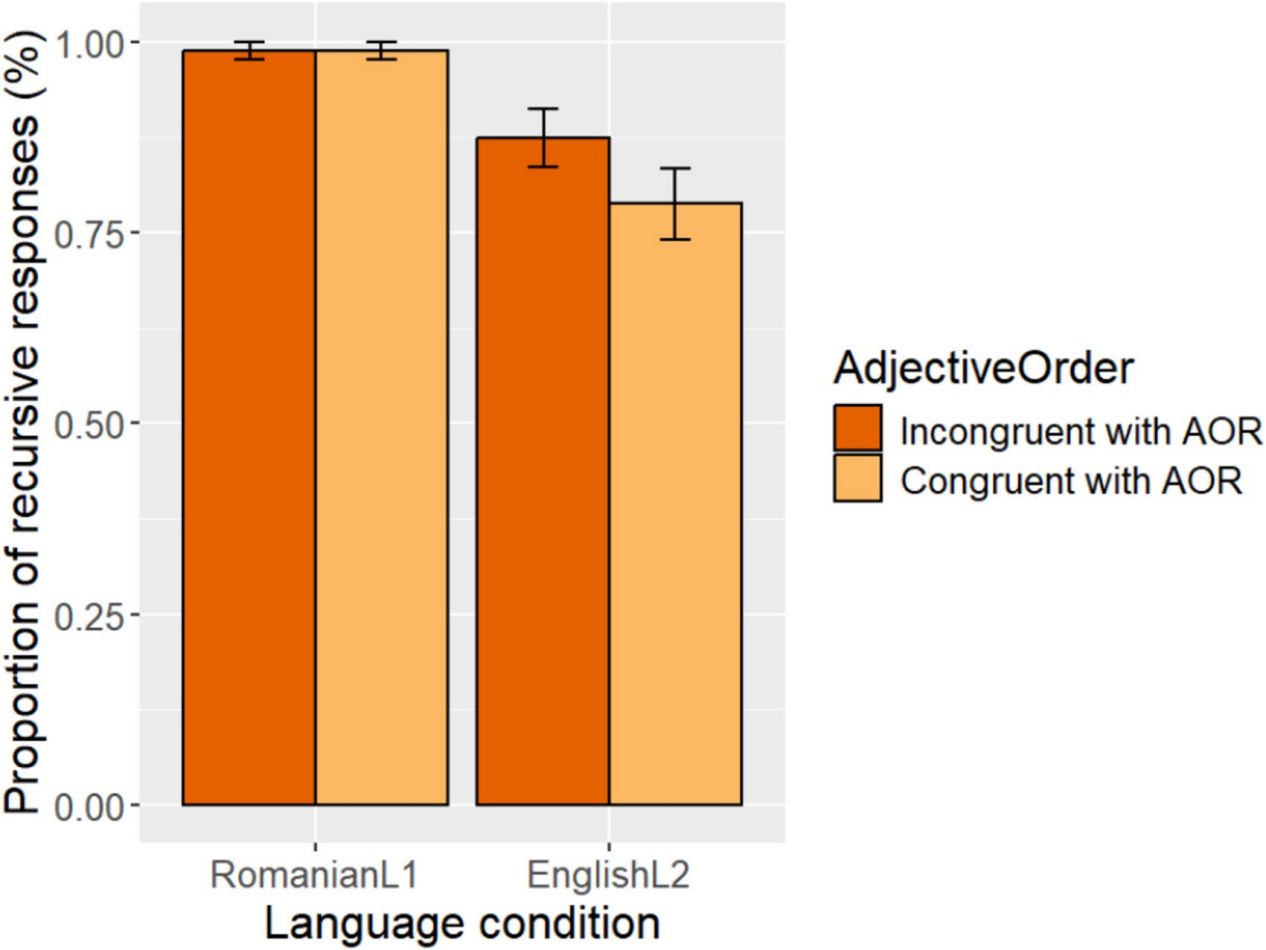
Proportion of target (accurate) recursive responses by Congruence condition Type in Romanian-L1 and English-L2.

Overall, the results indicated that both the Romanian-L1 group and the English-L2 group performed at a high level of accuracy, interpreting the adjectives recursively in each language (i.e., N A_set_ A_Subset_ in Romanian and A_Subset_ A_set_ N in English). Figure 2 shows the proportion of the target (accurate) recursive responses for each congruent condition for the two language condition groups. As shown in Figure 2, in the Romanian L1 group the proportion of the target recursive responses was 0.988 (*SD* = 0.109) in each of the two AOR congruent condition types: Congruent with AOR and Incongruent with AOR. In the English-L2 group, the proportion of the target recursive responses was 0.873 (*SD* = 0.335 in the Incongruent condition), which is somewhat numerically higher than the target recursive responses in the Congruent condition (0.788 with a *SD* of 0.412).

We employed a mixed effects logistic regression model. The dependent variable was Response, which was coded binomially (1 = target-like and as 0 = non-target-like). The fixed effects were Language Condition (Romanian L1 vs. English L2), AOR Congruence condition (Congruent/ Incongruent with AOR), and their interaction. The Model also employed by-participant slopes per AOR Congruence Condition type and random effects per Group. Alpha level was set at the 0.05 level of significance. The model showed that the interaction between Language Condition and AOR congruence condition type was not significant [*ß* = 1.064, *SE* = 1.788, *z* = 0.595, 95% CI (−2.440, 4.568), *p* = 0.552]. Nor was there any significant main effect for the AOR congruence condition [*ß* = −1.940e-06, *SE* = 1.690, *z* = 0.000, 95% CI (−3.324, 3.324), *p* = 0.999]. Only the main effect for the Language Condition was found to be significant, indicating that, when overall performance was considered (i.e., regardless of AOR Congruence Condition type), performance in English L2 was somewhat less RSSO-compliant than in Romanian L1 [*ß* = −4.123, *SE* = 1.863, *z* = −2.212, 95% CI (−7.774, −0.472), *p* = 0.027*].

In terms of non-target-like responses, we would like to highlight that in the Romanian L1 group, there was a total of 84 responses to the experimental items, and, of these, 2 (2.38%) were non-target answers (1 in the Congruent with AOR condition and 1 in the Incongruent with AOR condition). In the English L2 group, there was a total of 80 responses in all for the items in these two conditions. Of these responses, 28 (i.e., 35%) constituted non-target answers, with 10 non-target answers in the Incongruent with AOR condition and 18 non-target answers in the Congruent with AOR condition.

To determine whether participants consistently adhered to the RSSO, we used the following criteria. A participant was categorized as adhering to the RSSO if they provided target-like responses on at least 3 out of 4 trials in each of the two congruence with AOR condition types. Based on this criterion, 13/20 participants adhered to the RSSO in English-L2 (18/20 participants in the Incongruent with AOR condition type and 15/20 in the Congruent with AOR type) and 21/21 bilingual adults adhered to the RSSO in Romanian-L1 overall and in both condition types. A Fisher’s exact probability test was conducted to examine whether there were significant differences between the distribution of participants who met and did not meet the criteria in adhering to the RSSO across Romanian-L1 and English-L2. The test revealed that the two language groups differed significantly in relation to the distribution of RSSO-compliant and RSSO-non-compliant participants (*p* = 0.003448), with a greater likelihood of occurrence of RSSO-compliant participants in the Romanian-L1 language condition. Further comparisons with Fisher’s exact probability test carried out separately for each AOR congruence condition type (Congruent with AOR and Incongruent with AOR) showed that the differences in the distribution of RSSO-compliant and RSSO-non-compliant participants across the two language condition groups were statistically significant only in the case of the recursive adjective orders in the Congruent with AOR condition (*p* = 0.02069) but not in the case of the Incongruent with AOR condition (*p* > 0.05).

As mentioned above, 13 participants adhered to the RSSO in English L2, while 7 (i.e., 35% of the 20 participants in the English L2 group) did not. The errors produced by the 7 participants who failed to meet the criteria set for adherence to the RSSO were analyzed. The patterning of the errors is shown in Table 3. Regardless of the congruence condition type, the responses of two of the participants adhered to an adjectival ordering where the color adjective was the set (i.e., closest to the head noun) and the size adjective was further away from the noun (e.g., A_Size_ A_Color_ N, such as *small yellow leaves*). As can be seen from Table 3, one participant who showed context sensitivity interpreted adjectival sequences in an inverse manner instead, consistent with the postnominal L1 Romanian order. Thus, this participant associated the congruent A_Size_ A_Color_ N order with contexts where color subsets were picked from size sets, and the incongruent A_Color_ A_Size_ N order with contexts where size subsets were picked from color sets. The errors of the 4 remaining participants who did not consistently adhere to the RSSO, revealed a mixed patterning: 2 of them accepted both orders for contexts involving color subsets among size sets, while 2 others accepted both orders for contexts involving size subsets among color sets.

**Table 3:** Response patterns for the 7 participants who were not compliant with RSSO in English L2.

RSSO-non-compliant response type in L2	Only A_Size_ A_Color_ N	A_set_ A_subset_ N	Mixed (2 set-subset answers/2 non-set-subset answers)
Number of participants	2	1	4
Possible sources	L2 AOR Coordination	L1-based RSSO interpretation	L1-based RSSO L2 AOR Coordination

## 7 Discussion

According to our ***prediction in terms of UG***, we expected that bilinguals, in particular highly proficient bilinguals, will adhere to RSSO in both their L1 and L2, given that the Recursive Set Subset Ordering Constraint (RSSO) is a UG principle. This prediction was confirmed by our findings. Bilinguals did indeed show sensitivity to whether an adjective denotes a set or subset in both their first (L1) and second (L2) languages. This suggests that RSSO is available not only for the L1, but also for subsequent language acquisition. Importantly, regardless of the differences in branching directionality between languages (adjectives in Romanian are typically postnominal, whereas adjectives in English are typically prenominal), participants interpreted the adjective closest to the noun (N) as the set and the one further away from the head noun as the subset (98.8% answers were recursive in L1, 83.05% were recursive in L2). The high-performance levels in the two within subject conditions (i.e., orders congruent with AOR, incongruent with AOR) of the L1 Romanian condition is not at all surprising when we consider the long-term and in-depth immersion of the adult bilingual participants in an L1 Romanian environment (in both informal and formal contexts). The fact that the RSSO was adhered to not only in the L1, but also in L2 is remarkable, given that their dominant language was Romanian and more frequently used than English. The onset of exposure to English for our participants occurred around age 3–4. Crucially, they were children and not adults when they began their acquisition of L2 and this could play an important role in their ability to adhere to the RSSO in their L2 as well. Recall that Romanian employs the linear order Noun Set Subset, while English employs the linear order Subset Set Noun. The bilingual participants’ adherence to the RSSO across their two languages, regardless of the differences in branching directionality and linear order, highlights their ability to inhibit the activation of the other language when only one is in use. These findings provide support for activation of the RSSO universal constraint at the syntax-semantics interface across the two different L1 and L2 syntactic systems.

It is important to note that, in recursive set-subset contexts, their adherence to the RSSO takes precedence over Adjective Ordering Restrictions (AORs). Thus, while AORs can explain the linear order and interpretation of coordinative adjectives (where *small red flowers* is understood as ‘small and red flowers’), they cannot explain the recursive set-subset ordering and interpretation of recursive adjectives (where *red small flowers* is understood as the subset of red flowers within the set of small flowers). Even if conceptually, it has been argued that speakers may prefer placing the color adjective closer to the noun than the size adjective, most bilinguals in fact choose to interpret the adjective closest to the noun as the set even if it refers to the size when the contexts involve recursive set subsets.

As the results indicated, in the English L2 condition the proportion of target recursive interpretations was numerically lower in the Congruent with AOR condition. In contrast, in the Romanian L1 condition the proportion of target recursive interpretations was numerically equally high across the two Congruence with AOR conditions. Furthermore, the results of the Fisher’s test indicated significant differences between the L1 and L2 conditions only for the Congruent with AOR condition. These findings demonstrate that the participants experienced more difficulties in inhibiting the coordinative interpretation in the English L2 but not in their L1. It further demonstrates that in their English L2, participants had more difficulty with inhibiting a coordinative interpretation in the Congruent with AOR condition but not in the Incongruent with AOR condition type. One explanation for this result for English L2 is that in the Congruent with AOR condition the linear order (size color N) is consistent with the English AOR and is also consistent with the RSSO. However, in each case the interpretation is different. In the case of AOR, it can only be a coordinative interpretation that is not sanctioned by the context. In the case of the RSSO, the interpretation is a recursive set-subset one that is sanctioned by the set/subset context. Having two interpretations of the same linear order sequence of adjectives poses difficulties in processing. In contrast, the Incongruent Condition does not pose the same level of difficulty, as the target adjectival phrase (color size N) is not consistent with the AOR of English and can only be interpreted recursively. Here the coordinative interpretation would not be triggered automatically given that the linear order violates the AOR. Since there is no competing interpretation, participants would be able to direct their attention to the set/subset context in order to interpret the target adjectival phrase.

As noted previously, some researchers have proposed (contra Cinque) that sequential adjectives in Romanian may have a less rigid linear order in terms of AOR (the size adjective can precede the color adjective, *and* the color adjective can also precede size adjective). Interestingly, in the case of recursive adjectival contexts, the participants interpreted the adjective closest to the head noun as the set, and the adjective further away as the subset. Thus, the fact that bilinguals adhere to RSSO in both languages suggests that their sensitivity to this constraint is rooted in Universal Grammar (UG). Crucially, our findings indicate that they are not simply transferring the language-specific features of their L1 (i.e., post-nominal adjectival modifiers, and a more flexible AOR) into their L2. Instead, as proposed by Flynn et al. (2005), learners construct the new grammar via *grammatical mapping* from UG. The fact that most of the participants adhered consistently to the RSSO suggests that they were able to keep the L1 and L2 systems separate. Additionally, as illustrated in examples (1) and (2), English and Romanian differ in relation to morphological gender. English is a natural gender language, and Romanian is a language with grammatical gender, where nouns are categorized as masculine/feminine/neuter, and adjectives agree with the gender of the noun they modify. Such overt morphological differences between the two languages could have facilitated their maintenance as two separate systems. Crucially, this additional structural difference between Romanian L1 and English L2 may have made it easier for them to inhibit activation of the postnominal adjectival order of their dominant language, i.e., Romanian, when using English L2. Furthermore, their adherence to RSSO may have been facilitated by their native mastery of Romanian (L1) and their high (C1) proficiency in English (L2). Notably, our findings align with recent research showing that higher second language proficiency leads to a more native-like organization of semantics and semantic memory (see Kogan et al., 2024).

As for our ***prediction in terms of crosslinguistic influence***, given that the dominant language of Romanian L1-English L2 bilinguals is Romanian, we expected Romanian to influence their English to a greater extent. Overall, very few errors were attested in relation to recursive interpretation. The percentage of errors in the L2 English condition was 16.95%. In contrast, the percentage of errors in the L1 condition was negligible (1.2%), which suggests that, despite their high proficiency level in English, their L1 Romanian was impervious to influence from L2 English. Our findings suggest a slight influence of Romanian L1 on English L2 only in the case of the 7 participants who did not meet our criteria for adherence to the RSSO. A possible explanation is that for these 7 participants the adherence to RSSO may have been impacted by the inability to inhibit crosslanguage activation of language-specific features of their Romanian L1, leading to errors in the interpretation of recursive adjectives in L2.

The analysis of the 7 participants’ misinterpretations provides some insights into the mechanisms underlying the acquisition of recursive adjectival order in English L2. Recall that two participants consistently interpreted the prenominal color adjective as representing the set and the size adjective as representing the subset regardless of the condition. Under this view, these participants may be argued to have difficulty inhibiting a coordinate interpretation associated with the L2 AOR order. Such errors may have been further facilitated by prescriptive rules in formal instructional contexts English the correct ordering of adjectives is always *ASize AColor Noun*. Alternatively, another explanation for this error type is that it may be due to the existence of a universal semantic hierarchy of adjectives, where color properties, which are perceived as more objective than size properties (see Scontras et al., 2017, 2019), are somewhat more salient to participants, and tend to be favored as representing the set. As reported in the results section, one participant demonstrated sensitivity to the set-subset principle but interpreted adjectival sequences in the inverse order, in line with their L1 Romanian. Specifically, the size adjective was interpreted as the set in *ASize AColor* orders and the color adjective as the set in *AColor ASize* orders. This suggests failure to inhibit crosslanguage activation of L1, resulting in an interpretation based on a reversed direction of embedding. Recall that a mixed patterning was observed in the case of the errors made by four participants. Specifically, in at least one of the two congruence condition types, they provided the target recursive interpretation for 2 out of 4 trials and chose the non-target interpretation for the other 2 trials. We hypothesize that the mixed patterning of 2 non-target responses versus 2 target responses is not because of lack of L2 competence. On the contrary it may reflect difficulties in *performance* stemming from an inability to consistently inhibit L1 influence. An alternative explanation is that the four participants relied on a conjunctive default, treating the adjectives as coordinated (e.g., *small yellow flowers* interpreted as ‘small and yellow’). This strategy has been observed in previous research on child language acquisition in English and Romanian, where some children initially assign a conjunctive interpretation to recursive structures (e.g., *flori mici mari* ‘big small flowers’ interpreted as ‘big and small flowers’; see Bleotu and Roeper, 2021a, 2021b for Romanian; Foucault et al., 2021, 2022a, 2024 for English).

Recall that, when the distribution of the number of RSSO-compliant and RSSO-non-compliant participants was compared across the two language conditions using the Fisher’s test, significant differences were observed only in the Congruent with AOR condition, but not in the Incongruent with AOR type, with lower performance attested for English in comparison to Romanian. This can be explained in terms of failure on the part of a minority of participants to inhibit the L1 linear word order. Our overall findings, as well as the fine-grained analysis of the errors of the RSSO-non-compliant participants, fail to provide evidence in support of an L1 transfer based explanation, given that only one subject systemically showed difficulty in inhibiting the L1 pattern in L2.

Importantly, *grammatical mapping* from a UG-determined template (Lust, 2012; Berkes and Flynn, 2016; Fernández-Berkes and Flynn, 2023) helps account for our findings as to how Romanian L1-English L2 bilinguals are able to construct language-specific recursive set-subset structures. As discussed previously, Merge provides the universal minimalist template necessary for building hierarchical structure-dependent phrases. When interpreting recursive set-subset structures, participants take the adjective merged to the head noun first as the set and the adjective merged subsequently as the subset. They must, however, also integrate the branching directionality of the specific language in use (Romanian-right-branching, English-left-branching), ultimately assigning the interpretation Noun A_Set_ A_Subset_ in Romanian and A_Subset_ A_Set_ Noun in English. In our study, there were very few RSSO-non-compliant answers in English L2. Non-target response types involved: a coordinative interpretation, where the adjectives merge together first (Adj1^Adj2) before modifying the head noun, failure to inhibit the L1 recursive order or failure to inhibit the L2 AOR order. The very low frequency of occurrence of such non-target responses in our data, along with the overwhelming RSSO-compliant responses of most of the participants is in line with the cumulative enhancement model of acquisition (Flynn et al., 2004; Fernández-Berkes and Flynn, 2023).

One issue that needs to be discussed concerns the status of variability in our data, and whether it is sufficient to support our conclusions. As stated previously, one important goal of the current study was to compare how proficient Romanian L1-English L2 bilingual speakers, who were more dominant in their L1, observed the Recursive Set Subset Constraint across their two languages. Thus, we predicted that the group tested on L1 would perform at a very high level, while the group tested on L2 would experience some challenges in inhibiting the L1 influence. Importantly, the high-level performance of the group tested on L1 does not undermine the reliability or validity of the task. Rather, it serves to affirm the status of the Recursive Set Subset Ordering Constraint as a principle of Universal Grammar. In previous L1 research, there is overwhelming evidence in support of greater variability in children than in adult native speakers in relation to the RSSO constraint on adjectives (see Foucault et al., 2021, 2022a and Lakshmanan et al., 2024 for English children. See Bleotu and Roeper, 2021a, 2021b, 2022a, 2022b for Romanian children), as well as in relation to other recursive structures (Pérez-Leroux et al., 2017; Sevcenco and Avram, 2018; Giblin et al., 2019, and others). However, our focus here was on proficient Romanian L1-English L2 bilingual speakers. Additionally, the variability observed in the responses from the bilingual group tested on L2 is sufficient to shed light on the role of UG and cross-linguistic influence, given the existence of 7 participants who did not meet our criteria for target responses (representing 35% of the total number of 20 participants tested on L2). This ensures that the study captures individual variation and is not reliant on homogeneous groups. Thus, we can conclude that the RSSO-compliance of 13 participants (65%) in the English L2 condition compared to the overwhelming RSSO-compliance of all the participants in the Romanian L1 condition overwhelmingly asserts the validity of the universal status of the Recursive Set Subset Constraint. The fact that more non-target interpretations were attested in the L2 English condition than in the L1 Romanian condition points to a potential role for L1 in L2 processing.

Our data highlight a broader issue about how bilinguals navigate competing linguistic systems. At the outset of our paper, we mentioned that the term *crosslinguistic influence* rather than *transfer* better captures the interaction between two languages in the bilingual mind from a processing perspective. Acquisition of a new language is a creative process that does not simply entail transfer of language-specific constructions. Our overall findings clearly show that Romanian L1 - English L2 participants consistently observed the RSSO in English L2 regardless of language-specific differences in branching directionality between L2 and L1. In our English L2 data, of the 7 participants who did not consistently observe the RSSO, only one participant consistently exhibited difficulty in inhibiting L1 branching directionality when giving the target set-subset interpretation, while the evidence from the remainder of participants does not support L1 transfer. Instead, a few participants sometimes fail to inhibit L1 or competing coordinative L2 AOR orders, and this interferes with their interpretation. Overall, the crosslinguistic influence supports cumulative enhancement (Flynn et al., 2004; Fernández-Berkes and Flynn, 2023), as very few coordinative interpretations were observed. In other words, having acquired the RSSO in a previous language enables them to acquire their L2 more easily. Interestingly, if any form of transfer may be said to be at work, it is the transfer of a minimalist UG template, not language-specific structures.

Additionally, a few words are in order regarding the theoretical implications of our findings for syntactic accounts of RSSO in terms of Roll-Up and Adjunction. Interestingly, the fact that only 2 non-target responses were attested in Romanian L1, but more non-target responses (28, i.e., 35%) were attested in English L2 is compatible with both theoretical positions as long as we consider that English is the participants’ L2. If we assume, as in Roll-Up, that the default UG order is the prenominal adjectival order (as in English), and that the post-nominal adjectival order of Romanian is derived from this order via syntactic movement, then the higher rate of errors in English may seem unexpected given that bilinguals may be argued to have access to UG, and the English order is considered a default. However, it could nonetheless be explained through their greater familiarity with Romanian L1 and with the non-dominant status of L2. Thus, in spite of the higher syntactic complexity involved in deriving the L1 order, because participants have already established it as the order in their dominant language, they make fewer errors in L1. On the other hand, if we assume that both prenominal and postnominal orders are UG-consistent underlying orders (adjectives are adjoined to the right in Romanian but to the left of the head noun in English), the 7 participants’ difficulty with the prenominal order could again be explained through their challenges with L2, and their greater familiarity with L1. Thus, our results are in effect compatible with both theoretical positions (Roll-Up, Adjunction). Importantly, our paper makes a proposal regarding the universality of the RSSO without committing to a specific implementation (Roll-Up vs. Adjunction). What is crucial here is that the merging of the adjectives is hierarchically constrained by the set-subset adjective type, with set adjectives being merged to the head noun first and subset adjectives being merged subsequently, regardless of branching directionality.

Additionally, cross-linguistic variation in adjective ordering flexibility likely influences these findings. Romanian, often considered more flexible in adjective order regarding conceptual properties such as color or size, may allow the RSSO pattern to more easily override AOR patterns. Therefore, it would be important to conduct similar studies in languages which differ in branching directionality where **both** instantiate less flexible AORs. This would help determine whether participants face greater challenges with RSSO in languages with less flexible AORs.

Finally, it is worth noting that in our study, the target recursive phrases were presented using a neutral intonation pattern, without any contrastive stress or special emphasis placed on either of the two modifying adjectives. In future research, it would be interesting to examine whether the use of a marked prosodic intonation would impact the interpretation of recursive set-subset adjectives, what this would entail in a stress-timed language (e.g., English) or syllable-timed language (e.g., Romanian), and how it would interact with branching directionality (head-first vs. head-last).

## 8 Conclusion

Our study represents the first investigation of how adult sequential bilinguals handle recursive adjectives in contexts involving sets and subsets in their two languages (Romanian L1-English L2). Given participants’ high accuracy with recursion in both languages, our findings highlight the idea that the Recursive Set Subset Ordering Principle constrains bilingual acquisition and use. Our findings also provide evidence of crosslinguistic influence for some participants, which we attribute to language-specific differences in terms of branching directionality, linear order, and Adjective Ordering Restrictions.

## Data Availability

The datasets presented in this study can be found online in the OSF repository at the following link: https://osf.io/34ch5/?view_only=44a0c2f41f1848bc8bc6bec72d2b83ac.

## Footnotes

^1^While we use the term “speakers” inclusively here, we wish to point out that this would be an important area of research for sign languages as well.

^2^Note that an alternative approach to the distinction between right- and left-branching languages is offered by Kayne’s (1994) Antisymmetry Theory of Syntax. On the basis of the Linear Correspondence Axiom (LCA), Kayne (1994) argues that syntactic structures are universally right-branching due to an underlying hierarchical structure. However, while this theory offers a uniform explanation of word order, it is challenged by empirical evidence from languages with left-branching.

^3^For instance, bilinguals may struggle with orders involving a noun and multiple adjectives in L2 when the ordering differs from their L1, as seen in French-English children (Nicoladis, 2006) and French-Spanish adults (Androutsopoulou et al., 2008).

^4^Importantly, the recursive interpretation entails the coordinative one, i.e., the subset of red flowers that are big consists of flowers that are both big and red, while the coordinative interpretation does not entail the recursive one. While *big red flowers* and *red big flowers* are identical under a symmetric coordinative interpretation, they are not under a recursive reading (*the big red flowers* is not the same as *the red big flowers*).

^5^We embrace the Common European Framework of Reference for Languages (CEFR), which involves six levels: the A1 elementary level, the A2 pre-intermediate level, the B1 intermediate level, the B2 upper intermediate level, the C1 advanced level, and the C2 proficient level. Levels are organized into three blocks: A or basic user, B or independent user and C or proficient user (see https://www.britishcouncil.ro/en/english/levels).

^6^The smaller number of males compared to females reflects the overall male-to-female enrollment ratio in the Faculty of Letters at the University of Bucharest.

^7^A reviewer questioned the small number of experimental items per condition (4). However, it is important to clarify that this is not a sentence acceptability judgment task but rather a story cum picture-based comprehension task. Crucially, the task is cognitively demanding, requiring participants to integrate and assess information from both the story and accompanying images for each item. While the number of experimental items may seem small, the complexity involved in arriving at the target interpretation must be considered in the experimental design. Notably, participants took an average of 15 min to complete the task. Increasing the number of experimental items would have heightened fatigue, potentially affecting performance. Note that one of our goals is to build upon this research and run this experiment using the same design with younger children, who are cognitively less mature than adults. This was also a factor that we weighed in when deciding upon the number of items.

^8^An important point raised by an anonymous reviewer was why we opted for such a small number of fillers instead of a number that at least equals the number of experimental items. A larger number of fillers may be desirable in tasks such as sentence acceptability tasks or online sentence processing tasks in order to conceal the purpose of an experiment (i.e., the target structure). In our study, however, while the task itself is straightforward, the complexity of the recursive structures renders the specific details of the experiment less transparent to the participants. This is also consistent with previous practices in language acquisition research, where including fewer fillers (33% of the 12 items) is adequate if the test items are complex. Furthermore, as noted in relation to the number of experimental items, increasing the number of fillers would have led to fatigue, possibly impacting participants’ recursive interpretation (see Arehalli and Wittenberg, 2021).

## References

[ref1] Abels K. Neeleman A. (2010). Linear asymmetries and the LCA. Syntax 12, 25–74. doi: 10.1111/j.1467-9612.2011.00163.x

[ref2] Ahn D. Ferreira V. S. (2024). Shared vs separate structural representations: evidence from cumulative cross-language structural priming. Q. J. Exp. Psychol. 77, 174–190. doi: 10.1177/17470218231160942, PMID: 36960936 PMC10712209

[ref3] Androutsopoulou A. Español–Echevarría M. Prévost P. (2008). “On the acquisition of the pre-nominal placement of evaluative adjectives in L2 Spanish. Paper presented at 10th Hispanic linguistics symposium, London, Ontario Canada, 19–22 October 2006” in Proceedings of the 10th Hispanic linguistics symposium. eds. Bruhn de Garavito J. Valenzuela E. (Somerville, MA: Cascadilla Press), 1–12.

[ref4] Arehalli S. Wittenberg E. (2021). Experimental filler design influences error correction rates in a word restoration paradigm. Linguist. Vanguard 7:20200052. doi: 10.1515/lingvan-2020-0052

[ref5] Avram M. (1997). Anglicismele în limba română. București: Editura Academiei.

[ref300] Avram L. Sevcenco A. Tomescu V. (2020). The acquisition of recursively embedded noun modifiers in Romanian by Hungarian-Romanian bilinguals. Bucharest Working Papers in Linguistics 22, 61–84. doi: 10.31178/BWPL.22.1.4, PMID: 17723070

[ref6] Berkes É. Flynn S. (2016). “Multi-competence and syntax” in The Cambridge handbook of linguistic multi-competence. eds. Cook V. Wei L. (Cambridge: Cambridge University Press), 183–205.

[ref7] Bernolet S. Hartsuiker R. J. Pickering M. J. (2007). Shared syntactic representations in bilinguals: evidence for the role of word-order repetition. J. Exp. Psychol. Learn. Mem. Cogn. 33, 931–949. doi: 10.1037/0278-7393.33.5.931, PMID: 17723070

[ref8] Berwick R. C. Pietroski P. Yankama B. Chomsky N. (2011). Poverty of the stimulus revisited. Cogn. Sci. 35, 1207–1242. doi: 10.1111/j.1551-6709.2011.01189.x, PMID: 21824178

[ref9] Bialystok E. (2001). Bilingualism in development: Language, literacy, and cognition. New York: Cambridge University Press.

[ref10] Bleotu A. C. Luciu A. (2024). How are size, age, shape and color adjectives ordered in English and Romanian? An experimental investigation. Bucharest Work. Pap. Ling. XXVI, 69–86. doi: 10.31178/BWPL.26.1.4

[ref11] Bleotu A. C. Roeper T. (2021a). “Small big flowers or small and big flowers? Simple is better and roll-up is too complex for Romanian 5-year-olds” in Proceedings of the 45th annual Boston University conference on language development. eds. Dionne D. Vidal Covas L.-A., vol. 6 (Somerville, MA: Cascadilla Press), 66–79.

[ref12] Bleotu A. C. Roeper T. (2021b). Roll-up is too complex for Romanian 5-year-olds: evidence from recursive adjectives. Proc. Linguist. Soc. Am. 6, 133–143. doi: 10.3765/plsa.v6i1.4953

[ref13] Bleotu A. C. Roeper T. (2022a). The recursive set-subset ordering restriction overrides adjective ordering restrictions: evidence from Romanian 4-year-olds and adults. In: Gong Y. Kpogo F.. Proceedings of the 46th Annual Boston University Conference on Language Development. Somerville, MA: Cascadilla Press, 62–75.

[ref14] Bleotu A. C. Roeper T. (2022b). Children are more sensitive to the recursive set-subset ordering than to adjective ordering restrictions. Proc. Linguist. Soc. Am. 7:5267. doi: 10.3765/plsa.v7i1.5267

[ref15] Chomsky N. (1971). Problems of knowledge and freedom. The Russell Lectures. New York: Pantheon.

[ref16] Chomsky N. (1980). Rules and representations. New York: Columbia University Press.

[ref17] Cinque G. (1994). “On the evidence for partial N-movement in the romance DP” in Paths towards universal grammar: Studies in honor of Richard S. Kayne. eds. Cinque G. Koster J. Pollock J. Y. Rizzi L. Zanuttini R. (Washington, DC: Georgetown University Press), 85–110.

[ref18] Cinque G. (2005). Deriving Greenberg’s universal 20 and its exceptions. Linguist. Inq. 36, 315–332. doi: 10.1162/0024389054396917

[ref19] Cinque G. (2010). The syntax of adjectives: A comparative study. Cambridge, MA: MIT Press.

[ref20] Cinque G. (2023). On linearization: Toward a restrictive theory. Cambridge, MA: MIT Press.

[ref21] Cook V. J. (1991). The poverty-of-the-stimulus argument and multicompetence. Second. Lang. Res. 7, 103–117. doi: 10.1177/026765839100700203, PMID: 40518871

[ref22] Cook V. J. (2002). “Background to the L2 user” in Portraits of the L2 user. ed. Cook V. (Bristol: Multilingual Matters), 1–28.

[ref23] Cook V. J. (2003). “Introduction: the changing L1 in the L2 user’s mind” in Effects of the second language on the first. ed. Cook V. J. (Bristol: Multilingual Matters).

[ref24] Cook V. J. (2012). “Multicompetence” in The encyclopedia of applied linguistics. ed. Cook V. J. (Hoboken, NJ: Wiley).

[ref25] Cornilescu A. Cosma R. (2019). Linearization of attributive adjectives in Romanian. Revue Roumaine de Linguistique 64, 307–323.

[ref26] Costa A. (2009). “Lexical access in bilingual production” in Handbook of bilingualism: Psycholinguistic approaches. eds. Kroll J. F. Groot A. M. B. (New York: Oxford University Press).

[ref27] Crain S. Nakayama M. (1987). Structure dependence in grammar formation. Language 63, 522–543. doi: 10.2307/415004

[ref28] De Groot A. M. B. Kroll J. F. (1997). Tutorials in bilingualism. Mahwah, NJ: Erlbaum.

[ref29] De Vincenzi M. (1991). Syntactic parsing strategies in Italian: The minimal chain principle. Dordrecht: Kluwer Academic Publishers.

[ref30] Dixon R. M. W. (1982). ‘Where have all the adjectives gone?’ and other essays in semantics and syntax. Berlin: Mouton de Gruyter.

[ref31] Epstein S. D. Flynn S. Martohardjono G. (1996). Second language acquisition: theoretical and experimental issues in contemporary research. Behav. Brain Sci. 19, 677–714. doi: 10.1017/S0140525X00043521

[ref32] Epstein S. Flynn S. Martohardjono G. (1998). “The strong continuity hypothesis: some evidence concerning functional categories in adult L2 acquisition” in The generative study of second language acquisition. eds. Epstein S. Flynn S. Martohardjono G. (Mahwah, NJ: Lawrence Erlbaum), 61–79.

[ref901] Falk Y. Bardel C. (2010). The study of the role of the background languages in third language acquisition. The state of the art. International Review of Applied Linguistics in Language Teaching, 48, 185–219. doi: 10.1515/iral.2010.009

[ref33] Fernández-Berkes É. Flynn S. (2023). “Grammatical mapping in L3 acquisition: a theory of development” in L3 development after the initial state. eds. Brown-Bousfield M. Flynn S. Fernández-Berkes E. (Amsterdam: John Benjamins Publishing), 8–28.

[ref34] Flynn S. (1987). A parameter-setting model of L2 acquisition: Experimental studies in anaphora. Dordrecht: Reidel.

[ref35] Flynn S. Foley C. Gair J. Lust B. (2005). *Developmental primacy of free relatives in first, second and third language acquisition: implications for their syntax and semantics*. Paper presented at Linguistic Association of Great Britain, Cambridge University.

[ref36] Flynn S. Foley C. Vinnitskaya I. (2004). The cumulative-enhancement model for language acquisition: comparing adults’ and children’s patterns of development. Int. J. Multiling. 1, 3–17. doi: 10.1080/14790710408668175

[ref37] Fodor J. D. Frazier L. (1978). The sausage machine: a new two-stage parsing model. Cognition 6, 291–325. doi: 10.1016/0010-0277(78)90002-1

[ref38] Foucault D. (2020). A simple logical argument to elucidate the syntax/semantic interface of recursive set/subset. Recursion Workshop: University of Massachusetts, Amherst MA.

[ref39] Foucault D. Bai B. Bleotu A. C. Lakshmanan U. Merritt E. Sybing R.. (2021). *Relative gradable adjective recursion is more challenging for acquisition than possessive recursion*. Talk at the workshop Recursion Across Languages. The Intricacies of Babel (online), UMass Amherst, University of Bucharest, 1–2 June 2021

[ref40] Foucault D. Bleotu A. C. Lakshmanan U. Merritt E. Sybing R. Roeper T. (2022a). Relative gradable adjective recursion such as small small big mushrooms is more challenging for children than possessive recursion such as the deer’s friend’s sister’s mushrooms. Proc. Linguist. Soc. Am. 7:5294. doi: 10.3765/plsa.v7i1.5294

[ref41] Foucault D. Bleotu A. C. Lakshmanan U. Roeper T. (2022b). *Relative gradable adjective recursion is more challenging for acquisition than possessive recursion in English*. In: Recursion across Languages: The Intricacies of Babel.

[ref42] Foucault D. Lakshmanan U. Bleotu A. C. Roeper T. (2024). *Development of small big ideas through scaffolding story contexts: evidence from set-subset recursive adjectives in child English*. Talk presented at 2024 LSA Annual Meeting, 4–7 January 2024.

[ref43] Francis W. S. (2009). “Bilingual semantic and conceptual representation” in Handbook of bilingualism: Psycholinguistic approaches. eds. Kroll J. F. De Groot A. M. B. (New York: Oxford Academic).

[ref44] Gallo F. Bermudez-Margaretto B. Shtyrov Y. Abutalebi J. Kreiner H. Chitaya T.. (2021). First language attrition: what it is, what it isn’t, and what it can be. Front. Hum. Neurosci. 15:388. doi: 10.3389/fnhum.2021.686388 PMC8452950 34557079

[ref45] Gass S. Selinker L. (2008). Second language acquisition: An introductory course. 3rd *Edn*. New York: Routledge.

[ref908] Giblin I. Zhou P. Bill C. Shi J. Crain S. (2019). The spontaneous emergence of recursion in child language. In Brown M. M. Dailey B. (Eds.), Proceedings of the 43rd Boston University Conference on Language Development (pp. 270–285). Somerville, MA: Cascadilla Press.

[ref46] Green D. W. (1998). Mental control of the bilingual lexicosemantic system. Bilingualism Lang. Cogn. 1, 67–81. doi: 10.1017/S1366728998000133

[ref47] Grosjean F. (2010). Bilingual life and reality. Cambridge, MA: Harvard University Press.

[ref48] Guijarro-Fuentes P. Judy T. Rothman J. (2009). “On transfer, proficiency and cross-individual/aggregate SLA differences: examining adjectival semantics in L2 Spanish” in Issues in L2 proficiency. ed. Bennati A. G. (London: Continuum), 233–253.

[ref49] Gürel A. (2008). Review article: research on first language attrition of morphosyntax in adult bilinguals. Second Language Res. 24, 431–449. doi: 10.1177/0267658308093611

[ref50] Hartsuiker R. Pickering M. Veltkamp E. (2004). Is syntax separate or shared between languages: cross-linguistic syntactic priming in Spanish-English bilinguals. Psychol. Sci. 15, 409–414. doi: 10.1111/j.0956-7976.2004.00693.x, PMID: 15147495

[ref51] Judy T. Guijarro-Fuentes P. Rothman J. (2008). “Adult accessibility to L2 representational features: evidence from the Spanish Judy DP” in Selected proceedings of second language research forum 2007. eds. Bowles M. Foote R. Perpiñán S. Bhatt R. (Somerville: Cascadilla Press), 1–21.

[ref52] Kayne R. (1994). The Antisymmetry of syntax. Cambridge, MA: MIT Press.

[ref53] Kim Y. McDonough K. (2008). Learners’ production of passives during syntactic priming activities. Appl. Linguist. 29, 149–154. doi: 10.1093/applin/amn004

[ref54] Kogan B. Agulla L. Dottori M. Amoruso L. Vivas L. García A. M. (2024). Do we mean the same? Semantic native-likeness in highly proficient second language users. Int. J. Biling. 1:136. doi: 10.1177/13670069241267136, PMID: 40518871

[ref55] Kremers J. M. (2003). The Arabic noun phrase: a minimalist perspective [PhD thesis]. Nijmegen: University of Nijmegen.

[ref56] Kroll J. F. Stewart E. (1994). Category interference in translation and picture naming: evidence for asymmetric connection between bilingual memory representations. J. Mem. Lang. 33, 149–174. doi: 10.1006/jmla.1994.1008

[ref57] Kroll J. F. Tokowicz N. (2009). “Models of bilingual representation and processing: looking back and to the future” in Handbook of bilingualism: Psycholinguistic approaches. eds. Kroll J. F. De Groot A. M. B. (New York: Oxford Academic).

[ref58] Kroll J. F. van Hell J. G. Tokowicz N. Green D. W. (2010). The revised hierarchical model: a critical review and assessment. Biling. Lang. Cogn. 13, 373–381. doi: 10.1017/S136672891000009X, PMID: 20676387 PMC2910435

[ref59] Lakshmanan U. (1994). Universal grammar in child L2 acquisition: Morphological uniformity and null subjects. Amsterdam: John Benjamins.

[ref60] Lakshmanan U. (2024). “Syntactic maintenance of Tamil relative clauses in multilinguals” in The Oxford handbook of Dravidian languages. eds. Ragavachari A. Narasimhan B. (New York: Oxford University Press). doi: 10.1093/oxfordhb/9780197610411.013.30

[ref61] Lakshmanan U. Foucault D. Roeper T. (2022). *Does branching directionality impact bilingual children’s understanding of recursive structures in their stronger language?* Poster presented at GALA 15, Goethe Institute, Frankfurt, Germany, 22–24 September 2022

[ref62] Lakshmanan U. Foucault D. Roeper T. (2024). “Does branching directionality impact children’s understanding of recursive structures in their stronger language?” in Empirical and theoretical approaches to language acquisition: A generative perspective. eds. Weicker M. Lemmer R. Listanti A. Grimm A. (Newcastle: Cambridge Scholars Publishing), 226–254.

[ref63] Lardiere D. (1998). Case and tense in the ‘fossilized’ steady state. Second. Lang. Res. 14, 1–26. doi: 10.1191/026765898674105303

[ref904] Leandro W. Amaral L. (2014). The interpretation of multiple embedded genitive constructions by Wapichana and English speakers. Revista Linguí̂stica, 10, 149–162.

[ref64] Leivada E. Westergaard M. (2019). Universal linguistic hierarchies are not innately wired: evidence from multiple adjectives. PeerJ 7:e7438. doi: 10.7717/peerj.7438, PMID: 31396461 PMC6679903

[ref905] Limbach M. Adone D. (2010). Language acquisition of recursive possessives in English. In Franich K. Iserman K. M. Keil L. L. (Eds.), Proceedings of the 34th Annual Boston University Conference on Language Development (pp. 281–290). Somerville, MA: Cascadilla Press.

[ref65] Loebell H. Bock K. (2003). Structural priming across languages. Linguistics 41, 791–824. doi: 10.1515/ling.2003.026, PMID: 40292255

[ref66] Lust B. (1983). “On the notion “principal branching direction.”” in Studies in generative grammar and language acquisition. eds. Otsu Y. Van Riemskijk H. Inoue K. Kamio K. Kawasaki K. (Tokyo: Tokyo Gakugei University).

[ref67] Lust B. (2012). “Tracking universals requires a grammatical mapping paradigm” in Linguists of tomorrow: Selected papers from the first Cyprus postgraduate conference in theoretical and applied linguistics. eds. Grohmann K. Shelkovaya A. Zoumpaulidis D. (Newcastle upon Tyne: Cambridge Scholars Publishing), 105–130.

[ref68] Lust B. Flynn S. Foley C. Henderson C. Gair J. (2025). The Acquisition of Relativization. Cambridge: Cambridge University Press.

[ref69] Lust B. Mangione L. (1983). The principal branching direction parameter constraint in first language acquisition of anaphora. In: Sells P. Jones C. Proceedings of the NELS 13. Amherst: University of Massachusetts Press, pp. 145–160.

[ref906] Nelson J. S. (2016). First and second language acquisition of recursive operations: Two studies (Doctoral dissertation). University of Massachusetts Amherst.

[ref70] Nicoladis E. (2006). Cross-linguistic transfer in adjective–noun strings by preschool bilingual children. Biling. Lang. Cogn. 9, 15–32. doi: 10.1017/S136672890500235X

[ref71] Pavlenko A. (2004). “L2 influence and L1 attrition in adult bilingualism” in First language attrition: Interdisciplinary perspectives on methodological issues. eds. Schmid M. Kopke B. Keijzer M. Weimar L. (Amsterdam: John Benjamins), 47–59.

[ref72] Pavlenko A. Jarvis S. (2002). Bidirectional transfer. Appl. Linguist. 23, 190–214. doi: 10.1093/applin/23.2.190

[ref73] Pérez-Leroux A. T. Pettibone E. Castilla-Earls A. P. (2017). Down two steps: are bilinguals delayed in the acquisition of recursively embedded PPs? Matraga 24, 393–416. doi: 10.12957/matraga.2017.28781

[ref74] Pérez-Leroux A. T. Roberge Y. Pettibone E. Castilla-Earls A. (2021). *What can recursion tell us about bilingualism (and vice versa…)?* Presented at the online workshop Recursion Across Languages: The Intricacies of Babel, 1–2 June 2021. Available online at: https://lls.unibuc.ro/wp-content/uploads/2021/05/Roberge_What-does-recursion-tell-us-about-bilingualism.pdf.

[ref902] Pienemann M. Di Biase B. Kawaguchi S. Håkansson G. (2005). Processability, typological distance and L1 transfer. In Pienemann M. (Ed.), Cross-linguistic aspects of Processablity Theory, 85–116. Amsterdam: John Benjamins. doi: 10.1075/sibil.30.05pie

[ref75] Roeper T. (2011). The acquisition of recursion: how formalism articulates the child’s path. Biolinguistics 5, 57–86. doi: 10.5964/bioling.8831

[ref76] Roeper T. Snyder W. (2004). *Recursion as an analytic device in acquisition*. LOT Occasional Series 3, pp. 401–408.

[ref77] Rothman J. Guijarro Fuentes P. Iverson M. Judy T. (2009). *Noun-raising and adjectival interpretative reflexes in the L2 Spanish of Germanic and Italian learners*. In: BUCLD 33: Proceedings of the 33rd Annual Boston University Conference on Language Development. Sommerville, MA: Cascadilla Press, pp. 444–455.

[ref78] Rothman J. Judy T. Guijarro-Fuentes P. Pires A. (2010). On the (un)-ambiguity of adjectival modification in Spanish determiner phrases: informing debates on the mental representations of L2 syntax. Stud. Second. Lang. Acquis. 32, 47–77. doi: 10.1017/S0272263109990258

[ref79] Salamoura A. Williams J. N. (2006). Lexical activation of cross-language syntactic priming. Biling. Lang. Cogn. 9, 299–307. doi: 10.1017/S1366728906002641

[ref80] Salamoura A. Williams J. N. (2007). Processing verb argument structure across languages: evidence for shared representations in the bilingual lexicon. Appl. Psycholinguist. 28:627. doi: 10.1017/S0142716407070348

[ref81] Schmid M. S. (2011). Language attrition. Cambridge: Cambridge University Press.

[ref82] Schmid M. S. Köpke B. (2017). The relevance of first language attrition to theories of bilingual development. Linguist. Approaches Biling. 7, 637–667. doi: 10.1075/lab.17058.sch

[ref83] Schwartz B. D. Sprouse R. A. (1996). L2 cognitive states and the full transfer/full access model. Second. Lang. Res. 12, 40–72. doi: 10.1177/026765839601200103

[ref84] Scontras G. Degen J. Goodman N. D. (2017). Subjectivity predicts adjective ordering preferences. Open Mind 1, 53–65. doi: 10.1162/OPMI_a_00005

[ref85] Scontras G. Degen J. Goodman N. D. (2019). On the grammatical source of adjective ordering preferences. Semantics Pragmatics 12, 1–21. doi: 10.3765/sp.12.7

[ref907] Sevcenco A. Avram L. (2018). On the comprehension of recursive nominal modifiers in child Romanian. In Gavarró A. (ed.), On the Acquisition of the Syntax of Romance, 259–278. Amsterdam/Philadelphia: John Benjamins.

[ref86] Sharwood Smith M. (2021). Language transfer: a useful or pernicious concept? Second. Lang. Res. 37, 409–414. doi: 10.1177/0267658320941035

[ref87] Shin J. A. Christianson K. (2009). Syntactic processing in Korean-English bilingual production: evidence from cross-linguistic structural priming. Cognition 112, 175–180. doi: 10.1016/j.cognition.2009.03.011, PMID: 19394593

[ref88] Sproat R. Shih C. (1991). “The cross-linguistic distribution of adjective ordering restrictions” in Interdisciplinary approaches to language. eds. Georgopoulos C. Ishihara R. (Dordrecht: Kluwer), 565–593.

[ref89] Stoichiţoiu-Ichim A. (2001). Vocabularul limbii române actuale. Dinamică, influenţe, creativitate. Bucureşti: Editura ALL.

[ref90] Stoichiţoiu-Ichim A. (2002). “Asimilarea împrumuturilor englezeşti: Aspecte actuale ale dinamicii sensurilor” in Aspecte ale dinamicii limbii române actuale. ed. Pană-Dindelegan G. (Bucureşti: EUB), 249–262.

[ref91] Stoichiţoiu-Ichim A. (2003). ““Romgleza”: opţiune personală sau efect al globalizării?” in Identitate românească şi integrare europeană. ed. Gabor G. (Bucureşti: Editura Ars Docendi), 95–103.

[ref92] Teruya H. Lakshmanan U. (2019). *The effects of syntactic priming on English-L2 production by Japanese-English bilinguals*. Unpublished manuscript, University of Oregon and Southern Illinois University Carbondale.

[ref903] Trușcă D.-G. Bleotu A. C. (2024). Adjective orders in English and Romanian: An experimental investigation. Bucharest Working Papers in Linguistics, 26, 43–68. doi: 10.31178/BWPL.26.1.3

[ref93] Weinreich U. (1953). Languages in contact. The Hague: Mouton.

[ref94] White L. (2003). Second language acquisition and universal grammar. Cambridge: Cambridge University Press.

