# Supplementary material for: The role of Universal Grammar and crosslinguistic influence in the interpretation of recursive set-subset adjectives in adult Romanian L1-English L2 bilinguals
Source: Front Hum Neurosci. 2025 Aug 18;19:1537488. doi: 10.3389/fnhum.2025.1537488 (PMC12400931; doi:10.3389/fnhum.2025.1537488)
Supplement: Supplementary file 1 [file Data_Sheet_1.pdf]

Supplementary Materials

Table 1. Examples of practice items

<table>
<tr><th>Practice item focused on color</th><th>Practice item focused on size</th></tr>
<tr><td colspan="2">Romanian</td></tr>
<tr><td>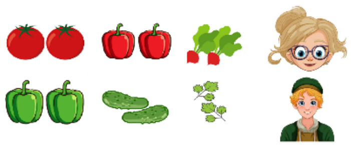

Cris şi Carol au fiecare legume în frigiderele lor. În frigiderul lui Chris, Cris are legume verzi. În frigiderul lui Carol, Carol are legume roşii. Pe cine ar trebui să rugăm să vedem roşiile?

Cris (Are legume verzi.)
Carol (Are legume roşii.)</td><td>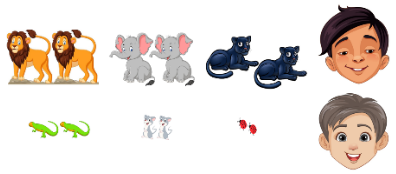

Toni şi Steve au fiecare animale în camerele lor. În camera lui Toni, Toni are animale mari. În camera lui Steve, Steve are animale mici. Pe cine ar trebui să rugăm să vedem șoarecii?

Toni (Are animale mari.)
Steve (Are animale mici.)</td></tr>
<tr><td colspan="2">English</td></tr>
<tr><td>Chris and Carol each have vegetables in their refrigerators. In Chris’s refrigerator, Chris has green vegetables. In Carol’s refrigerator, Carol has red vegetables. Who should we ask to see the tomatoes?

Chris (He has green vegetables.)
Carol (She has red vegetables.)

Expected answer: Carol</td><td>Toni and Steve each have animals in their rooms. In Toni's room, Toni has big animals. In Steve’s room, Steve has small animals. Who should we ask to see the mice?

Toni (He has big animals.)
Steve (He has small animals)

Expected recursive answer: Steve</td></tr>
</table>

Table 2. Examples of filler items

Filler item focused on color	Filler item focused on size
**Romanian**	
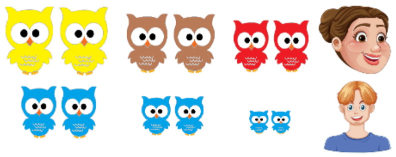<br>Lili şi Bill colecţionează fiecare bufniţe în casele lor. În casa lui Bill, Bill colecţionează bufniţe albastre. În casa lui Lili, Lili colecționează bufniţe mari. Pe cine ar trebui să rugăm să vadă *bufnițele mici*?<br><br>Lili (Are bufniţe mari.)<br>Bill (Are bufniţe albastre.)	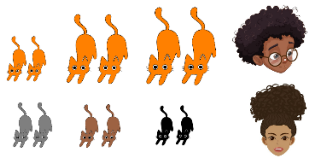<br>Mira şi Ben au fiecare pisicuță în curțile lor. În curtea Mirei, Mira are pisici mici. În curtea lui Ben, Ben are pisici portocalii. Pe cine ar trebui să rugăm să vedem *pisicile negre*?<br><br>Mira (Are pisici mici)<br>Ben (Are pisici portocalii)
**English**	
Lili and Bill each collect owls in their homes. In Bill's home, Bill collects blue owls. In Lili's home, Lili collects big owls. Who should we ask to see *the small owls*?<br><br>Lily (She has big owls.)<br>Bill (He has blue owls.)<br><br>Expected answer: ***Bill***	Mira and Ben each have kitties in their yards. In Mira's yard, Mira has small cats. In Ben's yard, Ben has orange cats. Who should we ask to see *the black cats*?<br><br>Mira (She has small cats.)<br>Ben (He has the orange cats)<br><br>Expected answer: ***Mira***
